# Parasitism of Locally Recruited Egg Parasitoids of the Fall Armyworm in Africa

**DOI:** 10.3390/insects11070430

**Published:** 2020-07-09

**Authors:** Saidou A. Laminou, Malick Niango Ba, Laouali Karimoune, Ali Doumma, Rangaswamy Muniappan

**Affiliations:** 1 International Crops Research Institute for the Semi-Arid Tropics (ICRISAT), BP 12404 Niamey, Niger; sayidelamine@gmail.com (S.A.L.); L.Karimoune@cgiar.org (L.K.); 2 University Abdou Moumouni, BP 10896 Niamey, Niger; doumma@yahoo.com; 3 Virginia Tech, 526 Price Fork Road, Blacksburg, VA 24061-0378, USA; rmuni@vt.edu

**Keywords:** *Telenomus remus*, augmentative release, biological control, trichogrammatid, *Spodoptera frugiperda*, fall armyworm, Niger, Sorghum

## Abstract

The fall armyworm (FAW), *Spodoptera frugiperda* (J.E. Smith) (Lepidoptera: Noctuidae), is an insect native to the tropical and subtropical Americas that has recently spread to Africa, where it predominately attacks maize, sorghum and other plant species. Biological control is an environmentally friendly way of combatting the pest and contributes to an integrated pest management approach. In Africa, several trichogrammatid parasitoids and *Telenomus remus* Nixon (Hymenoptera: Platygastridae) have been found parasitizing eggs of the FAW. In Niger, the egg parasitoids encountered include *Trichogrammatoidea* sp. (Hymenoptera: Trichogrammatidae) and *Telenomus remus* Nixon. Parasitism of the FAW eggs by the two egg parasitoids was assessed in the laboratory, followed by field testing on sentinel eggs. In the laboratory, *T. remus* parasitized on average 78% of FAW eggs, compared to 25% for *Trichogrammatoidea* sp. *Telenomus remus* was able to parasitize egg masses that were fully covered with scales, while *Trichogrammatoidea* sp. parasitized only uncovered egg masses. On-farm releases of *T. remus* in sorghum fields caused up to 64% of FAW egg parasitism. Parasitized eggs yielded viable progeny, which can contribute to FAW egg parasitism build-up during the cropping season. Our findings lay the groundwork for the use of *T. remus* in augmentative releases against FAW in Africa.

## 1. Introduction

The fall armyworm (FAW), *Spodoptera frugiperda* (J.E. Smith) (Lepidoptera: Noctuidae), is an insect native to the tropical and subtropical Americas that spread to Africa in early 2016 [1]. It became a serious pest mainly to maize, with early estimates in 2017 reporting yield losses of up 20.6 m tons in just 12 maize-producing countries [2]. Additionally, FAW attacks many other important food crops including sorghum and millet in Africa [3,4,5,6]. This damage has created problems all over Africa, leading to emergencies and responses by farmers with unproven efficacy, such as the application of ash, sand, botanical extracts, and other locally available materials [7]. Further, different governments procured synthetic pesticides, including highly toxic ones, and distributed them to the farmers [7,8]. To mitigate the risk of highly hazardous and broad-spectrum chemicals, alternate approaches are being developed, including plant-derived pesticides [9,10,11], habitat management practices through the use of companion cropping or push–pull technology [12,13], and biological control (BC) [14,15,16].

Biological control can offer an economical and environmentally friendly alternative for controlling FAW. In the Americas, BC is based on a variety of egg and larval parasitoids and predators [17,18,19,20,21]. The biological control approach contributes to an integrated pest management (IPM) strategy with other control means. As part of an IPM strategy, surveys of natural enemies of FAW were also conducted in several African countries [14,15,16,22,23]. These studies reported a wide range of larval parasitoids and egg parasitoids [14,15,16,22,23]. The egg parasitoid included *Telenomus remus* Nixon (Hymenoptera: Platygastridae) and several trichogrammatid species [15,16,22]. In Niger, the parasitoids encountered in maize and sorghum fields included several species belonging to the families Braconidae, Ichneumonidae, and Tachinidae, which parasitize larvae of FAW [24]. In addition, the two egg parasitoids *Telenomus* sp. and *Trichogrammatoidea* sp. were recorded [24]. *Telenomus* species was later identified as *Telenomus remus* Nixon [22]. *Trichogrammatoidea* sp. specimens (N°1569 NIGER Sadore 05-03-2019; NHMUK DNA21569/1 to NHMUK DNA21569/6) are permanently deposited in the Department of Life Sciences, Natural History Museum, London, UK [Polaszek A. prep]. *T. remus* is native to the Malay Peninsula and has been used in augmentative biological control (ABC) programs against *S. frugiperda* in the Americas [17,25,26,27,28]; however, since FAW and the parasitoid *T. remus* are being associated for the first time in Africa, it is necessary to assess the performance of this parasitoid before recommending its uses for ABC. Likewise, FAW and the parasitoid *Trichogrammatoidea* sp. are being associated for the first time; it is also worth assessing its performance while awaiting species identification. The first objective was to compare parasitism rates of FAW eggs between *T. remus* and *Trichogrammatoidea* sp. in the laboratory. Additionally, given that egg masses laid by FAW females are covered with scales from the female abdomen [25], we checked whether this feature affected parasitism by the two parasitoid species. The second objective was to test under field conditions, using sorghum plants infested with FAW egg masses, the parasitoid found to have a higher parasitism rate in the laboratory trials.

## 2. Materials and Methods

### 2.1. Study Environment

The lab bioassays and insect cultures were carried out in the entomology laboratory of the International Crops Research Institute for the Semi-Arid Tropics (ICRISAT) at Sadore in Niger (latitude 13°15′ N, longitude 2°18′ E) under a mean temperature of 25.60 ± 0.02 °C and mean relative humidity of 52.90 ± 0.16%. The on-station field releases experiment was conducted at the research campus of ICRISAT, Sadore. This agroecosystem has a unimodal rainfall pattern, with the rainy season extending from mid-May to mid-October. The field tests were carried out first in September 2019 toward the end of the rainy season and repeated in January 2020 after the rainy season. The average field temperature and relative humidity during FAW egg exposure and parasitoid releases were 31.23 ± 0.36 °C and 65 ± 1.75%, respectively, for the first field test. The average field temperature and relative humidity were 19.69 ± 0.10 °C and 17.48 ± 0.57%, respectively, for the second field test.

### 2.2. Insect Cultures for Bioassays

The insects mass-reared for this study included the FAW, *S. frugiperda*, the rice moth, *Corcyra cephalonica* Stainton (Lepidoptera: Pyralidae)*,* and the parasitoid wasps, *T. remus* and *Trichogrammatoidea* sp.

A colony of FAW was started from larvae collected in a sorghum field in late 2017 at the ICRISAT Research Station in Sadore, Niger. The insects were routinely reared in the laboratory. First instar larvae were fed with the tobacco budworm diet (Product# F9781B) from Frontier™ Agricultural Sciences, Newark, DE, USA. From second instars on, the larvae were exclusively fed on leaves of castor bean (*Ricinus communis* L.) using the method described by Prasanna et al. [29]. The FAW larvae were supplied fresh castor leaf, which was cut into small pieces. Three FAW larvae were put together in 36.96 ml plastic vials and supplied with 4.3 g of castor leaves. The leaves were replaced every 2–3 days depending on how long they remained green and fresh. The FAW larvae on the natural diet usually matured after 15–20 days, with a pupation period of 10 days. Moths of the FAW were given fresh leaves of sorghum to lay eggs in oviposition wire mesh cages (30 × 30 × 30 cm). Cotton wool soaked with sugar (10% sucrose in water solution) was hung in the cages to feed the moths. The leaves with attached eggs were cut and placed in egg hatching boxes. In about 4–6 days, the eggs developed into a blackhead stage and then hatched into neonate larvae. The neonate larvae were used to infest the artificial diet for the continuation of the colony. New wild FAW caterpillars were added to the colony in 2018 and 2019 to maintain insect vigor.

A colony of the rice moth *C. cephalonica* was established in the laboratory at ICRISAT, Sadore from wild insects collected in farmers’ granaries in Niger in 2015. The insects were routinely reared on a mixture of pearl millet grain and flour in plastic buckets at an ambient temperature [30]. Usually, adults emerged after one month. New wild adults were added to the colony every year to maintain insect vigor.

A colony of *Trichogrammatoidea* sp. was initially started from field-collected eggs of FAW in a sorghum field on the ICRISAT campus, Sadore in late 2018. The colony was maintained using the method described for the mass-rearing of related species *Trichogrammatoidea armigera* Nagaraja (Hymenoptera: Trichogrammatidae) [31]. Eggs were kept in Petri dishes in the laboratory under the above-mentioned temperature and humidity conditions until the emergence of adults. Emerging *Trichogrammatoidea* were collected daily, sexed, and placed in plastic tubes (ø = 2cm, h = 5.25 cm). The eggs of *C. cephalonica* were used to rear the parasitoid *Trichogrammatoidea* sp. The use of *C. cephalonica* eggs for rearing *Trichogrammatoidea* sp. was supported by early findings suggesting that *Trichogramma* ability to parasitize *S. frugiperda* was not affected once reared on related *Ephestia kuehniella* Zeller species [21,26,27]. The eggs of *C. cephalonica* were irradiated to halt their development and avoid the cannibalization of eggs by hatched larvae from unparasitized eggs as described earlier [31]. Irradiation of eggs was performed in a dark chamber under UV light 4 W tube (UVP, USA, 254 nm) for 45 min at a distance of 3 cm. *C. cephalonica* eggs were glued on white rectangular cards (4.5 cm × 1.75 cm), and a drop of honey was placed at the corner of the card to feed adult parasitoids. The culture was routinely maintained by the exposure of a first set of 30 eggs (1 day old) of *C. cephalonica* to a mated *Trichogrammatoidea* sp. female; this was followed by subsequent sets of 30 eggs (1 day old) every day until the death of the female. The females were provided a new male for mating every 3 days for 24 h. The average life span of *Trichogrammatoidea* sp. female was 11.34 ± 1.26 days; the development from egg to adult was completed in 9.65 ± 0.57 days. Each female produced approximately 120 adults in a female biased sex-ratio 1:2.

The rearing technique of *T. remus* is similar to *Trichogrammatoidea* sp. except FAW eggs were used instead of *C. cephalonica*. The colony of *T. remus* was initially started from field-collected parasitized eggs of FAW in a sorghum field at ICRISAT campus, Sadore in late 2018. Emerging adults were sexed and given egg masses of FAW collected from the mass-rearing colony. Leaves of sorghum bearing FAW egg masses were cut and glued onto a carboard sheet (7.3 cm × 4.2 cm), and a droplet of honey was placed at the corner of the card to feed *T. remus* adults. The egg cards were exposed for 2 days to a mated *T. remus* female in plastic flasks (ø = 4.5 cm; h = 11.5 cm) using a ratio of 20 eggs to 1 wasp. The female was given fresh FAW egg masses every 2 days to parasitize until her death. The average life span of the *T. remus* female was approximately 17.5 ± 2.21 days; the development from egg to adult was completed in 11.21 ± 0.63 days. Each female produced approximately 200 adults in a female-biased sex-ratio.

### 2.3. Assessment of FAW Egg Parasitism by T. remus and Trichogrammatoidea sp. in the Laboratory

This experiment was conducted using the fresh egg masses of FAW collected from a mass-rearing facility. In general, eggs laid by FAW females were covered with scales from the female abdomen [25], and as the female aged, egg masses were less or not covered [32]. The egg masses were split into three groups: (i) egg masses fully covered with scales, (ii) egg masses partially covered with scales, and (iii) egg masses without scales. In our rearing condition, each of the egg masses had roughly 100 eggs. The different egg masses were exposed to 24-h-old mated females of *T. remus*, *Trichogrammatoidea* sp., or combined *T. remus* + *Trichogrammatoidea* sp. for 24 h to allow oviposition. The experiment was carried out in three-factor (Egg scale, type of parasitism; egg scale × parasitism type interaction) factorial with 3 levels within each factor, and with 13 replicates. For each treatment, we used 24-h-old mated wasp females. The incubation of eggs and parasitoids were carried out in plastic vials (ø = 3 cm; h = 8.5 cm) for 24 h, and the parasitized eggs were incubated until the emergence of the new generation of parasitoids. Data on parasitism and emerging adults were recorded.

### 2.4. Assessment of Field Performance of Telenomus remus Following Augmentative Releases

#### 2.4.1. Preparation of the *T. remus* Cards for On-Station Field Releases

All *T. remus* parasitoids used in the releases were from laboratory colonies reared on FAW eggs. Parasitoid cards (7.3 cm × 4.2 cm dimension) were prepared 11 days prior to field releases. Each card had 14 irradiated egg masses of FAW (with ≈50 eggs in each) parasitized with 12 *T. remus* mated females for 48 h. *T. remus* adults began emerging from cards the day they were placed in the field, and after 2 days, all the parasitoids had emerged. Each card produced approximately 415 parasitoids (60% females). The parasitoid cards were placed individually in a thick envelope to protect them from sunlight, direct rainfall, and predation. The envelope was perforated to create small holes that allowed emerging *T. remus* adults to disperse.

All *T. remus* parasitoids used in the releases were from laboratory colonies reared on FAW eggs. Parasitoid cards (7.3 cm × 4.2 cm dimension) were prepared 11 days prior to field releases. Each card had 14 irradiated egg masses of FAW (with ≈50 eggs in each) parasitized with 12 *T. remus* mated females for 48 h. *T. remus* adults began emerging from cards the day they were placed in the field, and after 2 days, all the parasitoids had emerged. Each card produced approximately 415 parasitoids (60% females). The parasitoid cards were placed individually in a thick envelope to protect them from sunlight, direct rainfall, and predation. The envelope was perforated to create small holes that allowed emerging *T. remus* adults to disperse.

#### 2.4.2. On-Station Releases

This experiment was performed first in September 2019 and repeated in January 2020. The experiment was carried out on sorghum. For each of the two trials, a local popular sorghum variety named *Hankorin karuwa* (with an 85 day maturing cycle, sensitive to FAW) was planted in a spacing arrangement of 1 m × 1 m. For each trial, the sorghum was planted in eight fields of 200 m^2^ each with 400 sorghum plants at 200 stands (two plants/stand). All consecutive fields were separated by 200 m of grassland. The eight fields were divided into two groups of four: (i) four release fields that were each supplied with *T. remus* parasitoid, and (ii) four control fields that did not receive any parasitoids. Within each of the eight fields, one plant in every two stands was infested with FAW egg masses. This corresponds to 100 sorghum plants infested in each plot (25%). The plants were infested 2 weeks after planting using the sentinel egg technique [20,33,34]. Freshly laid egg masses of FAW were removed from sorghum leaves in the laboratory culture, taken to the field, and pasted to the upper face of sorghum leaves with nontoxic glue to mimic naturally laid eggs. The egg masses were covered with a thin mesh (ø= 1 mm) that prevented the predation of eggs but allowed parasitism by parasitoids. Each selected plant was infested with one egg mass of ≈50 eggs.

The infestation of sorghum with FAW eggs occurred on the same day as the placement of parasitoid cards. The parasitoids were released once at different points of the field using cards with parasitized FAW eggs on the day *T. remus* adults began emerging. The envelopes bearing the parasitoid cards were fastened to a sorghum stalk at 0.5 m above ground at three points of the field. All the parasitoids typically emerged in 2 days. No parasitoids were released in any of the control fields. The *T. remus* parasitoid was released at the rate of 15 *T. remus* females per 100 FAW eggs as recommended in other settings [35]. This corresponded to 1250 parasitoids per 200 m^2^ (750 females and 500 males) and three parasitoid cards per release field. Egg masses that were placed in both release and control fields were recollected 4 days after exposure and taken back to the laboratory for incubation at ambient temperature and the observation of parasitism, emergence of parasitoids, and dead eggs. Emerging parasitoids were identified and counted.

### 2.5. Data Analysis

The percentage parasitism was computed by calculating the ratio of the total number of parasitized eggs to the total of eggs counted within an egg mass. The percentage of emerging parasitoids was calculated as the ratio of the total number of emerging adults to the total of parasitized eggs. The assumptions of normality of residuals was confirmed using the Shapiro–Wilk test. The data were subjected to arcsine transformation prior to ANOVA with SAS software version 9.1 [36]. Laboratory data were subjected to two-way ANOVA to compare parasitoid species (3 levels) and egg mass type (3 level) and their interactions. Likewise, field data of all years were combined together and subjected to two-way ANOVA to compare treatments and years and their interactions. Means were separated by Bonferroni post hoc test.

## 3. Results

### 3.1. Parasitism of FAW Eggs by T. remus and Trichogrammatoidea sp. in the Laboratory

**Figure 1:**
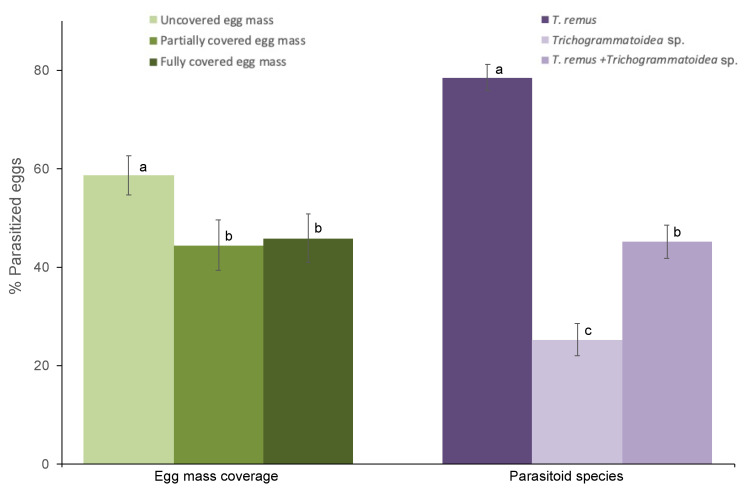
Egg parasitism of *Spodoptera frugiperda* (% ± SE) by *T. remus*, *Trichogrammatoidea* sp.; and combined *T. remus* + *Trichogrammatoidea* sp. of fully covered, partially covered, and uncovered egg masses. Bars bearing different letters within column groups were significantly different (Bonferroni test, α = 0.05).

The egg mass coverage significantly affects the parasitism regardless of species (F_2,106_ = 5.67; *p* = 0.005). Likewise, the parasitism was significantly affected by parasitoid species regardless of egg mass type (F_2,106_ = 74.07; *p* < 0.001). However, the egg mass coverage and parasitoid species interaction does not affect the parasitism (F_4,106_ =2.18; *p* = 0.07). The parasitism on uncovered egg masses was significantly higher than the two other egg masses (Figure 1). The egg parasitism by *T. remus* was significantly higher than the parasitism by *Trichogrammatoidea* sp. and combined *T. remus* + *Trichogrammatoidea* sp. regardless of egg mass type (Figure 1).

**Figure 2:**
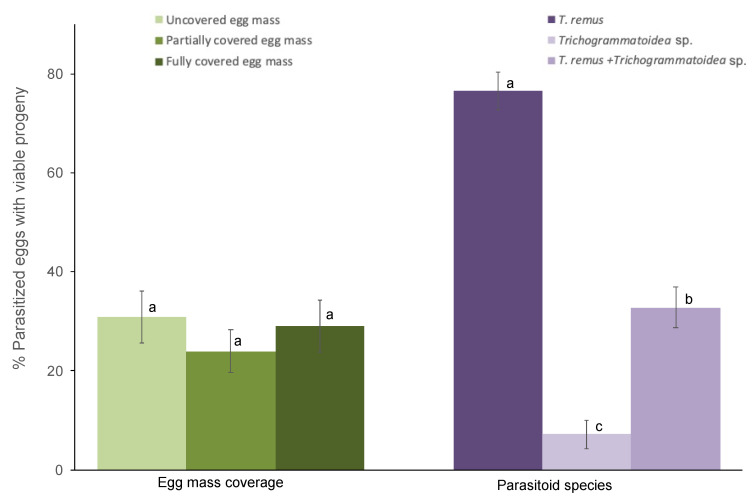
Viable progeny emerging from eggs of *Spodoptera frugiperda* (% ± SE) parasitized by *T. remus*, *Trichogrammatoidea* sp., and combined *T. remus* + *Trichogrammatoidea* sp. of fully covered, partially covered, and uncovered egg masses. Bars bearing different letters within column groups were significantly different (Bonferroni test, α = 0.05).

The egg mass coverage does not affect parasitoid emergence regardless of species (F_2,102_ = 1.55; *p* = 0.21; Figure 2). However, progeny emergence varied by parasitoid species (F_2,102_ = 84.37; *p* < 0.001). The interaction between parasitoid species and egg mass coverage does not affect emergence (F_4,102_ = 1.04; *p* = 0.39). Eggs subjected to *T. remus* parasitism yielded significantly more progeny than combined *T. remus* + *Trichogrammatoidea* sp. or *Trichogrammatoidea* sp. alone regardless of egg mass type (Figure 2). Emerging progeny from combined parasitism included *T. remus* (97%) and *Trichogrammatoidea* sp. (3%).

### 3.2. Parasitism of T. remus Following on-Station Releases

**Figure 3:**
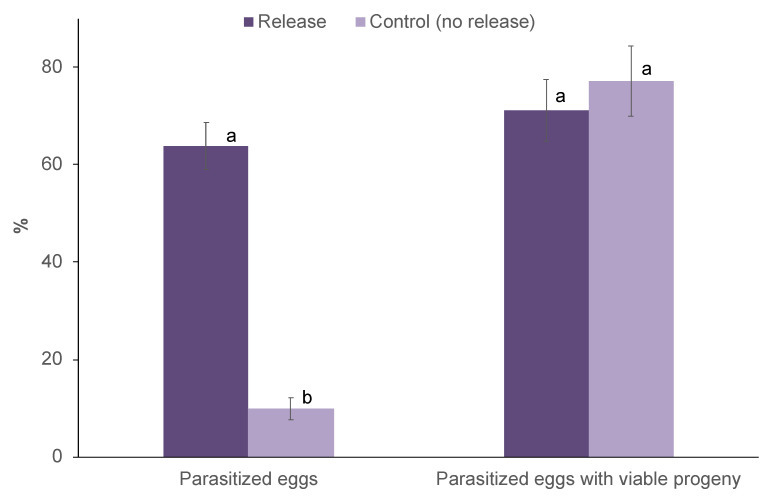
Parasitized eggs of *Spodoptera frugiperda* (% ± S.E.) in release and control fields following on-station augmentative releases of *T. remus* and corresponding emerging progenies (% ± S.E.). For each parameter, treatments bearing different letters were significantly different (Bonferroni test, α = 0.05).

The overall parasitism for both trials was significantly higher in released fields than control fields (F_1,12_ = 93.99; *p* < 0.001; Figure 3). However, a significant difference was not found for years (F_1,12_ = 1.59; *p* = 0.23). Likewise, the years and treatments interaction does not affect the parasitism (F_1,12_ = 0.29; *p* = 0.60). 

For both trials, up to 77% parasitized eggs yielded viable *T. remus* progeny, with no significant difference between released and control fields (F_1,10_ = 1.01; *p* = 0.34; Figure 3). Likewise, a significant difference was not found for years (F_1,10_ = 0.04; *p* = 0.85). However, the years and treatments interaction affect parasitoids emergence (F_1,10_ = 7.32; *p* = 0.02). Emerging progeny included *T. remus* (95%) and *Trichogrammatoidea* sp. (5%) in both released and control fields in September 2019, while in the January 2020 trial, only *T. remus* progeny emerged from parasitized eggs of both treatments.

## 4. Discussion

In the laboratory, *T. remus* parasitized significantly more FAW eggs than *Trichogrammatoidea* sp. Unlike *Trichogrammatoidea* sp., *T. remus* was able to overcome the layer of scales on egg masses and parasitize all types of egg masses equally. This is consistent with previous findings [37,38]. *T. remus* was described as an aggressive parasitoid due to its larger, more robust size (as compared to *Trichogrammatoidea*), which enables it to penetrate all layers of the egg mass of FAW and parasitize more eggs [25]. Similar observations were made for the related species *Telenomus nawai* Ashmead on the eggs of related host species *Spodoptera litura* Fabricius [39]. In our experimental conditions, *T. remus* parasitized on average 78% of FAW eggs in the laboratory. This is below the 100% FAW egg parasitism reported in other settings for *T. remus* [25,37]. The discrepancies may be due to laboratory conditions, as the performance of *T. remus* is affected by temperature and humidity [40,41,42,43]. Differences may also be explained by parasitoid strains [35], as well as the egg mass/parasitoid ratio [44] and exposure time.

Usually, the presence of scales over FAW egg masses constitutes a barrier against parasitism by *Trichogrammatidae* [45,46]. It is thus not surprising that *Trichogrammatoidea* sp. did not parasitize more than 25% of FAW eggs, with most parasitized eggs being those that were not covered by the protective scale. However, this level of parasitism by *Trichogrammatoidea* sp. is very low compared to *Trichogramma pretiosum* Riley (Hymenoptera: Trichogrammatidae), a parasitoid used in the biological control of FAW in Nicaragua [21]. In fact, *T. pretiosum* also avoids egg masses with too many scales, parasitizing only the upper layer of egg masses and those found on the edge of the egg masses, which can be easily reached from the side [47]. The poor performance of *Trichogrammatoidea* sp. on *S. frugiperda* could be due to an early learning experience as the parasitoid was mass reared on *C. cephalonica* before being given *S. frugiperda* eggs. Similar behavior has been reported with some *Trichogramma* species in other settings [48,49,50]. The poor performance of *Trichogrammatoidea* sp. on *S. frugiperda* could also be due to the size of the rearing eggs. In fact, eggs of *C. cephalonica* are smaller than those of *S. frugiperda* [51], and as reported in other settings, once reared in small eggs trichogrammatid could be smaller and this could affect the parasitism [52,53,54,55,56]. Further studies are, however, needed to reach conclusions as with *T. pretiosum*, its mass rearing on *Ephestia kuehniella* eggs does not affect its performance once releases in the field against FAW [21].

Given that *T. remus* emergence always occurred at the range of one parasitoid per host egg [25,40,57], and that *T. remus* parasitism was higher than that of *Trichogrammatoidea* sp., a higher emergence of *T. remus* adults when both parasitoids were put together in the same arena could have been expected. Unfortunately, the contrary was observed, meaning that the combination of *T. remus* and *Trichogrammatidae* sp. adversely affected the overall parasitism of FAW eggs. In fact, the interaction of different parasitoid species can lead to contrasting results [58,59,60,61,62]. This could be due to exploitative competition toward eggs of FAW and/or interference competition, as observed between *Trichogramma pretiosum* Riley, *Trichogramma atopovirilia* Oatman & Platner and *T. remus* [34]. In fact, by being quicker in ovipositing and searching for FAW eggs, *T. remus* likely parasitized a larger number of eggs within a shorter period of time than its competitor [47], and thus subjected itself to superparasitism by *Trichogrammatoidea* sp., as suggested by previous studies [34]. Similar findings were observed for *T. pretiosum* on the related *Telenomus heliothidis* Ashmead species [63]. Further studies are, however, needed to reach conclusions on *Trichogrammatoidea* sp. and *T. remus* interactions. Although overall parasitism is affected, significantly higher numbers of *T. remus* emerged from parasitized eggs than *Trichogrammatoidea* sp. This reflects a competitive dominance of *T. remus* over *Trichogrammatoidea* sp. Similar findings have been reported for *T. remus* over *Trichogramma atoporivilia* and *Trichogramma pretiosum* [34,47], and for related *Telenomus ullyetti* Nixon over *Trichogrammatoidea lutea* Girault [64].

From the field experiments, releases of *T. remus* led to significantly higher parasitism of FAW eggs compared to control fields that did not receive parasitoids. Nevertheless, our study showed positive behavior characteristics for *T. remus* as a biological control agent of FAW in a sorghum cropping system. Interestingly, released *T. remus* were able to find and parasitize sentinel host eggs within the sorghum field. Similar findings were reported with *T. remus* in corn cropping systems infested with FAW in the Americas [25,26,28,34,37]. In our case, the augmentative release of *T. remus* resulted in 64% parasitism compared to 60%–90% in Latin America [25,26,37]. Discrepancies in parasitism could be due to differences in the host egg/parasitoid ratio, as reported earlier [28,65]. Furthermore, we used artificially infested plants, while previous studies operated under naturally infested crops. As observed in other settings, sentinel eggs could dramatically underestimate actual rates of parasitism by several parasitoid species [66,67], including the related species, *Telenomus podisi* Ashmead [68]. In fact, *T. remus* respond to a combination of kairomones including those present in the secretion of the *S. frugiperda* female accessory gland [69] and the sex pheromone of *S. frugiperda* [70]. Moreover, volatiles emitted by plants attacked by FAW caterpillars in the canopy can serve indirectly as cues for locating inconspicuous host stages, such as eggs by *T. remus* as reported in other settings [71]. In our case, both plant volatiles and sexual pheromones might be missing as wild FAW females and crop damage were not observed in the sorghum field during the experiment. All these considerations in combination could explain the lower *T. remus* parasitism in our experimental conditions. Another factor that could influence *T. remus* parasitism is the host crop. Most of the studies into *T. remus* parasitism on FAW are from maize cropping systems, while we used sorghum. As reported earlier, the performance of *T. remus* is affected by its host crop [65,72,73]. In fact, for several parasitoid species, the performance is habitat-specific [74,75,76,77,78]. Other factors such as temperature and humidity could explain the differences in parasitism [38,40,43,73,79].

The importance of parasitoid emergence after on-farm releases must also be considered because it can compromise the maintenance of the parasitoid in the field. This is particularly necessary in an environment in which farmers plant at different times in the season and FAW lays eggs over an extended period of time, and when several generations of FAW could develop in the cropping season. *T. remus* progeny emerging from the first batch of released parasitoids can eventually contribute to the control of subsequent generations of FAW. As indicated by Ferrer [26], *T. remus* parasitism increased over weeks. In our case, the emergence of *T. remus* adults from parasitized eggs in the field was low compared to other findings, and this could be due to temperature. As reported by Bueno et al. [40], suitable temperatures for higher viability are between 20–31 °C. Above 35 °C, no *T. remus* adults emerged from parasitized eggs. In our case, the parasitized eggs were exposed to variable field temperatures for four days, which could reach 38 °C at some points of the day. Similarly, when taken back to the laboratory for incubation, parasitized eggs experienced ambient temperature, which was also fluctuating. All these factors in combination could explain the discrepancies in the percentage of emergences. Interestingly, *Trichogrammatoidea* sp. adults emerged from sentinel egg masses of FAW subjected to *T. remus* augmentative releases in September 2019. This indicates that FAW eggs were also parasitized by naturally occurring trichogrammatids. A similar observation was reported in Brazil [28], and the researchers there also found significantly more parasitism due to released *T. remus* than naturally occurring by trichogrammatids. However, in January 2020 in the dry season, no *Trichogrammatoidea* sp. emerged from parasitized eggs, suggesting a seasonal occurrence of the parasitoid. These findings confirm the natural occurrence of *Trichogrammatoidea* sp. on eggs of FAW in Niger [24]. In other regions of Africa, trichogrammatid egg parasitoids were only encountered from the *Trichogramma* genus [15,16].

## 5. Conclusions

This study showed that augmentative releases of the egg parasitoid *T. remus* significantly increase the parasitism of FAW eggs in sorghum fields. This finding lays the groundwork for the use of *T. remus* in biocontrol programs in an integrated approach for controlling FAW in Africa. Further on-farm assessment is being conducted to identify the exact numbers needed per crop acreage in relation to *T. remus* dispersal. Likewise, the rearing of *T. remus* on an alternative host, *Corcyra cephalonica* Stainton, should be considered for cost-effective mass production, as suggested by Viera et al. [80].
